# Reduced cortical thickness in patients with acute-on-chronic liver failure due to non-alcoholic etiology

**DOI:** 10.1186/s12967-015-0679-6

**Published:** 2015-10-06

**Authors:** Santosh K. Yadav, Rakesh K. Gupta, Vivek A. Saraswat, Murali Rangan, Michael A. Thomas, Sergio Rutella, Silvio Danese, Ena Wang, Francesco M. Marincola, Mohammad Haris

**Affiliations:** Research Branch, Sidra Medical and Research Center, P.O. Box 26999, Doha, Qatar; Department of Radiology, Fortis Memorial Research Institute, Gurgaon, Haryana India; Department of Gastroenterology, Sanjay Gandhi Post Graduate Institute of Medical Sciences, Lucknow, India; Department of Radiological Sciences, David Geffen School of Medicine at University of California at Los Angeles, Los Angeles, California USA; Department of Gastroenterology, IRCCS Humanitas Research Hospital, Milan, Italy

**Keywords:** Acute-on-chronic liver failure, Hepatic encephalopathy, FreeSurfer, Cortical thickness, Magnetic resonance imaging

## Abstract

**Background:**

Acute-on-chronic liver failure (ACLF) is a form of liver disease with high short-term mortality. ACLF offers considerable potential to affect the cortical areas by significant tissue injury due to loss of neurons and other supporting cells. We measured changes in cortical thickness and metabolites profile in ACLF patients following treatment, and compared it with those of age matched healthy volunteers.

**Methods:**

For the cortical thickness analysis we performed whole brain high resolution T1-weighted magnetic resonance imaging (MRI) on 15 ACLF and 10 healthy volunteers at 3T clinical MR scanner. Proton MR Spectroscopy (^1^H MRS) was also performed to measure level of altered metabolites. Out of 15 ACLF patients 10 survived and underwent follow-up study after clinical recovery at 3 weeks. FreeSurfer program was used to quantify cortical thickness and LC- Model software was used to quantify absolute metabolites concentrations. Neuropsychological (NP) test was performed to assess the cognitive performance in follow-up ACLF patients compared to controls.

**Results:**

Significantly reduced cortical thicknesses in multiple brain sites, and significantly decreased N-acetyl aspartate (NAA), myo-inositol (mI) and significantly increased glutamate/glutamine (glx) metabolites were observed in ACLF compared to those of controls at baseline study. Follow-up patients showed significant recovery in cortical thickness and Glx level, while NAA and mI were partially recovered compared to baseline study. When compared to controls, follow-up patients still showed reduced cortical thickness and altered metabolites level. Follow-up patients had abnormal neuropsychological (NP) scores compared to controls.

**Conclusions:**

Neuronal loss as suggested by the reduced NAA, decreased cellular density due to increased cerebral hyperammonemia as supported by the increased glx level, and increased proinflammatory cytokines and free radicals may account for the reduced cortical thickness in ACLF patients. Presence of reduced cortical thickness, altered metabolites and abnormal NP test scores in post recovery subjects as compared to those of controls is associated with incomplete clinical recovery. The current imaging protocol can be easily implemented in clinical settings to evaluate and monitor brain tissue changes in patients with ACLF during the course of treatment.

## Background

Acute-on-chronic liver failure (ACLF) is a form of liver disease resulting in acute deterioration of liver function in patients with chronic liver disease and subsequently multiple organ failure over a period of weeks, and high short term mortality [1–3]. The prevalence of non-alcoholic related ACLF is not well documented, however, it is reported that 9–32 % of Indian population have non-alcoholic related fatty liver diseases with higher frequency in obese and diabetic patients, which is suggestive of higher prevalence of ACLF in non-alcoholic liver diseases [4, 5]. ACLF often presents with liver failure as the first evidence of liver disease, manifested by serious complications and, subsequently, by hepatic encephalopathy (HE) [6, 7]. This syndrome is generally thought to result from neuronal dysfunction, diminished cerebral metabolism, morphological changes in astrocytes, and neuronal loss, leading to functional deficits of varying severity ranging from subtle minimal changes to severe coma [8–12].

ACLF offers considerable potential to affect**s** the cortical areas by significant tissue injury due to loss of neurons and other supporting cells. Except for the nature of tissue changes and brain metabolites alteration, regional cortical thickness integrity in ACLF patients is unknown. Previously, reduced gray matter density and cortical thickness have been reported in chronic liver disease patients with minimal HE and history of overt HE [10, 13–17]. So far, no study is available in other forms of liver failure such as in ACLF with severe HE. Precise determination of regional cortical thicknesses changes may assist evaluation of processes underlying the deficient functional characteristics in ACLF patients.

Neuroimaging and proton MR spectroscopy (^1^H MRS) techniques have been used to characterize the brain tissue injury and altered cerebral metabolites in patients with ACLF [18–21]. A majority of patients with HE generally show no visible abnormalities on conventional magnetic resonance imaging (MRI), except for hyperintensities on T1-weighted images in the deep gray matter due to deposition of manganese [22–24]. On diffusion tensor imaging (DTI) studies, these patients have shown reduced fractional anisotropy (a marker of microstructural tissue integrity), and altered mean diffusivity (indicator of water diffusion), in multiple brain areas, suggestive of the presence of abnormal tissue structures and cerebral edema [18]. On ^1^H MRS significantly decreased *N*-acetyl aspartate (NAA), myoinositol (mI) and increased glutamate/glutamine (Glx) levels were observed in these patients [19]. Higher Glx levels in brain is associated with increased brain’s glutamine concentration which results from detoxification of ammonia and is associated with neuropsychological changes [25], while NAA is a marker of neuronal density/function, and mI provides information about glial cells activation/proliferation [26].

Voxel based morphometric analyses have been used to quantify the brain’s structural changes in various pathologies. The regional gray matter volume and tissue integrity assessed with voxel-based morphometric analysis may be influenced by analytical differences in extra-cortical cerebrospinal fluid and surface curvature/complexities. An alternative approach is to examine regional cortical thicknesses, which can be performed with specialized software designed to quantify the regional cortical thickness changes, and such assessment is provided by FreeSurfer software [27].

In the current study, we aim to evaluate the regional cortical thicknesses and brain metabolites profiles in patients with ACLF in comparison with those of normal volunteers. We also assess the effect of conservative treatment on cortical thickness and metabolites profile after clinical recovery in these patients. Since these patients have increased blood ammonia level and high oxidative stress due to end stage liver disease that can act as a neurotoxin, we hypothesized that reduced cortical thickness and brain metabolites level would appear in ACLF over control subjects, especially in the cortical areas serving autonomic, motor, and cognitive functions, and that will improve after clinical recovery.

## Methods

### Participants

Institutional ethical committee approved the study protocols. Informed consent was obtained from the nearest kin of all patients, as well as from individual controls, after explaining the nature and purpose of the MRI study. Fifteen patients with ACLF (males/females; 12/3, mean age 42 ± 8.4 years) and 10 age/sex matched healthy controls (males/females; 8/2, mean age 39 ± 13.0 years) underwent brain MRI, clinical and biochemical examination. Out of 15 patients, 10 patients underwent repeat brain MRI and cognitive assessment after clinical recovery at 3 weeks of conservative treatment and were considered as clinically recovered. Demographic and clinical data of ACLF patients are summarized in Table 1. ACLF was diagnosed when there was evidence of an acute hepatic insult manifesting as jaundice and coagulopathy, complicated within 4 weeks by ascites and/or encephalopathy in a patient with previously diagnosed or undiagnosed chronic liver disease [28]. West Haven criteria were used to grade the HE in these patients [29]. In order to establish the presence, severity, etiology and complications of the liver disease, blood tests including differential blood counts, liver and renal function tests, ascitic fluid examination, serologic tests for hepatotropic viruses, investigations for metabolic liver diseases and other investigations were performed. The exlusion criteria were as follows: patients with prior porta-systemic shunt surgery, prior neurological or psychiatric illness, active use of medications for psychiatric illnesses, significant alcohol intake (>40 gm/day in men, >20 gm/day in women) over 6 months prior to imaging, ultrasonographic evidence of a mass lesion in the liver suggestive of hepatocellular carcinoma, biliary obstruction or suspected sepsis at presentation and unsatisfactory image acquisition caused by motion artifacts. Both ACLF and control subjects were also excluded from the study if they were claustrophobic, or carried non-removable metal, such as braces, embolic coils, pacemakers/implantable cardioverter-defibrillators, stents.

**Table 1:** Characteristics and outcome of patients with acute-on-chronic liver failure

Cases	Age/sex	Chronic etiology	Acute etiology	Grade of HE	Jaundice-HE interval (days)	Outcome
#1	45/F	Cryptogenic	HEV	2	3	Discharged
#2	41/M	Cryptogenic	HEV	2	4	Discharged
#3	48/M	Cryptogenic	HEV	2	4	Discharged
#4	50/M	Wilson disease	ATT	4	2	Discharged
#5	50/M	HBV	HBV	1	25	Discharged
#6	44/M	Cryptogenic	Unknown	2	20	Discharged
#7	30/M	HBV	HBV	2	4	Discharged
#8	35/F	Cryptogenic	HBV	3	4	Discharged
#9	50/F	Cryptogenic	HAV	3	3	Discharged
#10	50/M	HBV	HBV	2	2	Deceased
#11	32/M	HCV	HEV	4	9	Deceased
#12	35/M	Cryptogenic	HEV	4	14	Discharged
#13	25/M	Cryptogenic	Unknown	4	23	Deceased
#14	47/M	Cryptogenic	Unknown	3	12	Deceased
#15	48/M	Cryptogenic	Unknown	1	14	Deceased

*HBV* hepatitis B virus, *HEV* hepatitis E virus, *HCV* hepatitis C virus, *HE* hepatic encephalopathy

Neuropsychological (NP) test was performed to evaluate cognitive functions in post recovery ACLF patients compared to controls. We performed a well-established trial making test and Wechsler Adult Intelligence Scale (WAIS)-P tests on these subjects, which can assess visual motor coordination, concentration, mental speed, memory and attention. We were unable to perform NP test at baseline study because these patients were encephalopathic and unconscious.

### Management protocol

All patients were managed according to a standard protocol, which includes close monitoring and meticulous optimization of all metabolic derangements [30]. Oxygenation was continuously monitored in each patient using pulse-oximeter and the blood glucose level was monitored 6-hourly using a glucometer. Vital parameters, neurological status and signs of raised intracranial pressure (ICP) were monitored 4-hourly. Aggressive correction of any hypoglycemia, hyponatremia or hypokalemia was performed according to standard guidelines. Patients with ACLF who had clinical evidence of raised ICP received intravenous mannitol at a dose of 1–1.5 gm/kg/24 h, divided into four boluses, for up to 48–72 h.

### Magnetic resonance imaging data acquisition

All patients and controls underwent whole brain MRI on a 3.0 Tesla MR scanner (HDXT, Signa, General Electric technologies Systems, Milwaukee, WI) using an 8-channel head coil. Patients transported for MR imaging accompanied with a staff trained in resuscitation, critical care and with life-support facilities on standby and ventilation support were started if they were unable to maintain atrial PaO2 >60 mmHg or SaO2 >90. None of the patients received any kind of sedation and/or muscle paralyzing agents during imaging. Routine brain MRI studies including fast spin echo T2-weighted images with repetition time (TR)/echo time (TE)/number of excitations (NEX) = 5660 ms/98 ms/3, and T1-weighted fast spin echo images with TR/TE/NEX = 1950 ms/8.4 ms/1 were performed. High-resolution T1-weighted brain images were also acquired using a fast spoiled gradient recalled echo (FSPGR) pulse sequence (TR = 8.4 ms; TE = 3.32 ms; inversion time = 400 ms; flip angle = 13°; matrix size = 512 × 512; field of view = 240 × 240 mm^2^; slice-thickness = 1.0 mm) from each subject. ^1^H MRS was performed using single voxel point resolved spectroscopy (PRESS) method with following parameters—TR/TE = 3000 ms/35 ms and number of averages = 64. A voxel of 2 × 2 × 2 cm^3^ was placed mainly in the right basal ganglion region of the brain in all subjects. Both T1-, and T2-weighted images were examined for any gross brain pathology such as cysts, tumors, or any other mass lesions, and presence of such anomaly was used as an exclusion criteria. None of the patients and control subjects included in this study showed any major pathology.

### Measurements of cortical thicknesses

High-resolution T1-weighted brain images were used to measure the regional cortical thicknesses in all subjects by applying cross sectional pipeline of FreeSurfer (v 5.3.0), as described in detail elsewhere [27]. Briefly, high-resolution T1-weighted brain images volumes from each subject were converted into FreeSurfer data format. Data processing included removal of non-brain tissue using a hybrid watershed/surface deformation procedure, automated Talairach transformation, intensity normalization, segmentation of sub-cortical white and deep gray matter tissue types, tessellation of the gray and white matter boundaries, automated topology correction, and surface deformation (http://surfer.nmr.mgh.harvard.edu/). To measure changes in the cortical thickness between baseline and follow-up scans of ACLF patients, images were processed using the FreeSurfer longitudinal pipeline [31] (http://surfer.nmr.mgh.harvard.edu/fswiki/LongitudinalProcessing). All subjects’ processed data were manually evaluated to ensure no brain areas were excluded. Similarly, gray, white, and pial boundaries were visually assessed, and if needed, edits were made to correct misidentified regions. In most subjects, only minor edits were required to remove non-brain areas after automatically detected skull strip procedures.

### ^1^H-MR spectroscopy data processing

For evaluation and quantification of all individual spectra, the LC- Model software package (Version 6.0) was used [32]. The absolute concentration of NAA, Glx, and mI were calculated.

### Statistical analysis

#### Demographic and clinical variables

IBM Statistical Package for the Social Sciences (IBM SPSS, v 16, Chicago, IL, USA) was used for statistical analyses. Subjects’ demographic, clinical, and neuropsychological profiles were assessed by independent samples t-tests and Chi square. One-way analysis of variance (ANOVA) was performed to compare ^1^H MRS derived metabolites levels in different study groups. A p < 0.05 value was considered statistically significant.

#### Regional cortical thicknesses

Gray matter surface maps were smoothed using a Gaussian kernel (FWHM, 15 mm). Regional changes in cortical thicknesses among different groups were examined using a vertex-by-vertex general linear model, implemented in FreeSurfer, with regional cortical thicknesses modeled as a function of groups, and age and gender included as covariates in the analysis (ANCOVA; p < 0.05, false discovery rate corrections for multiple comparisons). The statistical parametric maps with regional cortical thickness differences were generated separately for the left and right hemispheres. Clusters with significant differences between groups were overlaid onto averaged inflated cortical surface maps for structural identification.

## Results

### Demographics

No significant differences were observed either in gender (p = 0.50) or age distribution (p = 0.38) among groups.

### Clinical profile of ACLF patients

Clinical and biochemical profile of ACLF patients is summarized in Table 1. The etiology of acute components in 15 patients with ACLF was hepatitis A (n = 1), hepatitis B (n = 4), hepatitis E (n = 5), antituberculosis drugs (n = 1) and unknown (n = 4), and the etiology of chronic liver disease was hepatitis B (n = 3), hepatitis C (n = 1), Wilson disease (n = 1), and cryptogenic (n = 10). At the time of imaging, 4 patients were in grade IV, 3 patients in grade III, 6 patients in grade II and 2 patients in grade I encephalopathy (Table 1).

### Cortical thicknesses findings in controls, baseline and post recovery ACLF patients

**Fig. 1:**
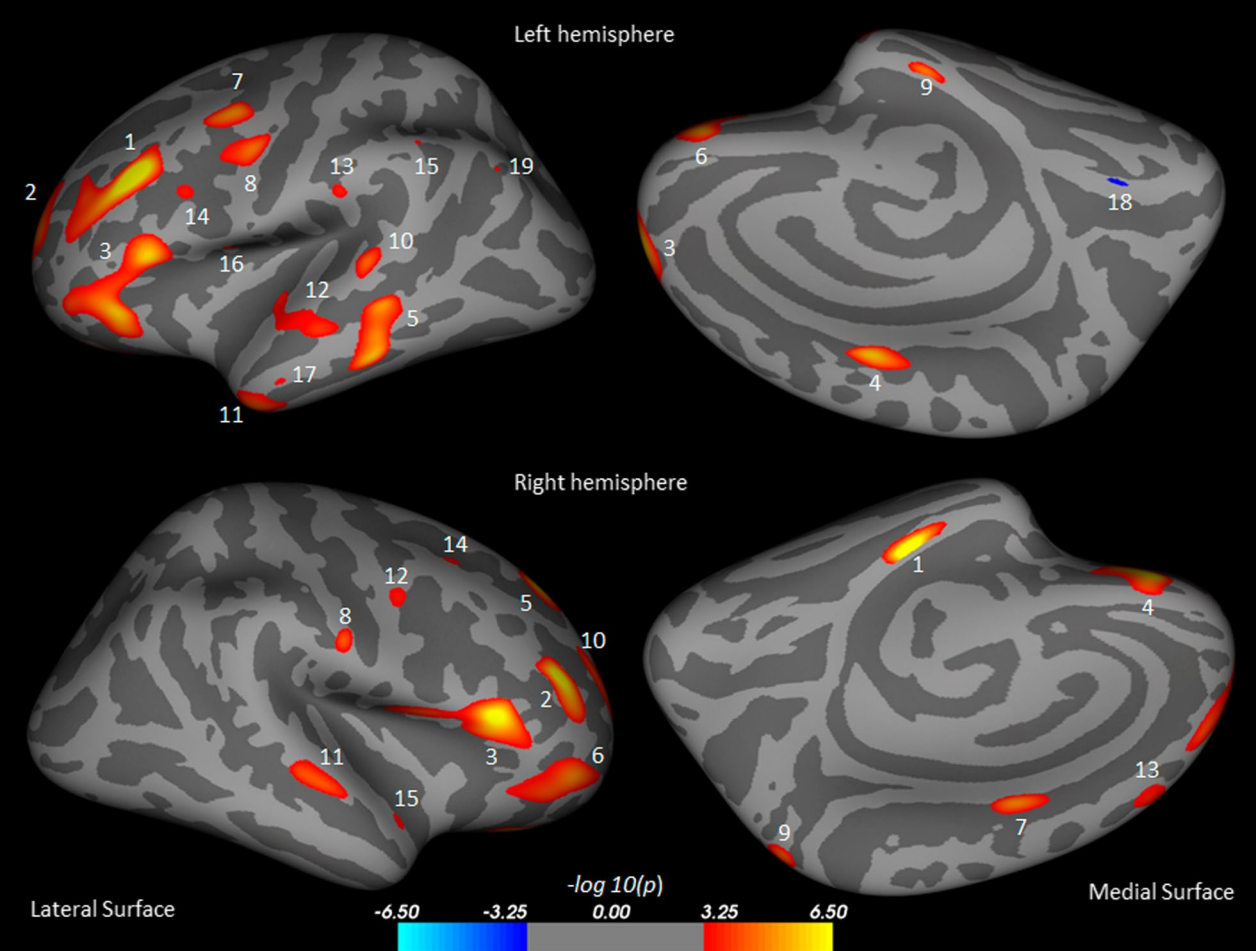
Brain regions showing significantly reduced cortical thickness in baseline ACLF patients compare to control subjects in the left hemispheres overlaid on inflated pial surface, include rostral middle frontal (*1*), superior frontal (*2*), pars triangularis (*3*), posterior cingulate (*4*), inferior temporal (*5*), lateral orbitofrontal (*6*), caudal middle frontal (*7*), precentral (*8*), entorhinal (*9*), superior temporal (*10*), inferior temporal (*11*), middle temporal (*12*), supramarginal (*13*), pars opercularis (*14*), supramarginal (*15*), pars opercularis (*16*), middle temporal (*17*), lingual (*18*), and inferior parietal (*19*) and right hemispheres include parahippocampal (*1*), rostral middle frontal (*2*), pars triangularis (*3*), lateral orbitofrontal (*4*), superior frontal (*5*), pars orbitalis (*6*), posterior cingulate (*7*), postcentral (*8*), precuneus (9), rostral middle frontal (*10*), superior temporal (*11*), precentral (*12*), superior frontal (*13*), caudal middle frontal (*14*), and superior temporal (*15*)

At baseline study, ACLF patients showed significantly decreased cortical thickness in multiple brain areas both in the left and right hemisphere compared to those of controls. Regions with decreased cortical thickness in the left hemisphere were rostral middle frontal, superior frontal, pars triangularis, posterior cingulate, inferior temporal, lateral orbitofrontal, caudal middle frontal, precentral, entorhinal, superior temporal, inferior temporal, middle temporal, supramarginal, pars opercularis, supramarginal, pars opercularis, middle temporal, and inferior parietal (Table 2; Fig. 1), while in the right hemispheres these regions were- parahippocampal, rostral middle frontal, pars triangularis, lateral orbitofrontal, superior frontal, pars orbitalis, posterior cingulate, postcentral, precuneus, rostral middle frontal, superior temporal, precentral, superior frontal, caudal middle frontal, superior temporal (Table 2; Fig. 1). Only the lingual region in the left hemisphere showed increased cortical thickness.

**Table 2:** Surface areas of significantly reduced cortical thickness in baseline ACLF over control from both hemispheres

Cluster no.		Structures in area	Max t-statistic	Size (mm^2^)	TalX	TalY	TalZ	Cortical thickness (Mean ± SD)	
								Control	Baseline
1	L	Rostral middle frontal	6.48	965	−42.7	29.1	30.1	2.45 ± 0.10	2.17 ± 0.11
2	L	Superior frontal	4.55	935	−9.6	63.2	13.7	2.66 ± 0.24	2.24 ± 0.16
3	L	Pars triangularis	4.52	1251	−50.8	24.9	5.9	2.58 ± 0.13	2.29 ± 0.12
4	L	Posterior cingulate	4.41	162	−10.9	−2.6	39.8	2.60 ± 0.13	2.35 ± 0.17
5	L	Inferior temporal	4.31	536	−55.5	−33.2	−16.6	2.76 ± 0.17	2.48 ± 0.11
6	L	Lateral orbitofrontal	4.16	284	−12.4	42.6	−21.9	2.16 ± 0.17	1.89 ± 0.11
7	L	Caudal middle frontal	3.95	264	−42	2.4	48.5	2.65 ± 0.23	2.32 ± 0.13
8	L	Precentral	3.67	229	−55.1	−3.2	38.8	2.69 ± 0.14	2.46 ± 0.10
9	L	Entorhinal	3.59	75	−19.9	−14.2	−28.5	3.16 ± 0.18	2.80 ± 0.20
10	L	Superior temporal	3.51	137	−63.5	−30.1	7.3	2.68 ± 0.15	2.40 ± 0.15
11	L	Inferior temporal	3.51	454	−41.2	−0.2	−36.8	3.30 ± 0.25	2.81 ± 0.26
12	L	Middle temporal	2.99	390	−51.2	−20.2	−14.8	2.64 ± 0.25	2.33 ± 0.13
13	L	Supramarginal	2.80	35	−60	−26.8	21.2	2.54 ± 0.17	2.38 ± 0.13
14	L	Pars opercularis	2.76	29	−34.7	12.7	24.4	2.22 ± 0.17	2.01 ± 0.10
15	L	Supramarginal	2.63	11	−52.2	−47	43.9	2.63 ± 0.18	2.38 ± 0.16
16	L	Pars opercularis	2.59	15	−44.2	5.2	7.8	2.67 ± 0.15	2.49 ± 0.15
17	L	Middle temporal	2.59	18	−56.4	−7.9	−27.8	3.08 ± 0.24	2.78 ± 0.17
18	L	Lingual	−2.58	21	−7.4	−68.3	−0.3	1.71 ± 0.13	1.97 ± 0.23
19	L	Inferior parietal	2.54	5	−39.7	−68.5	35.1	2.45 ± 0.12	2.23 ± 0.17
1	R	Parahippocampal	5.74	191	23.3	−23.6	−23.2	2.93 ± 0.17	2.48 ± 0.16
2	R	Rostral middle frontal	5.29	537	35.9	45.7	21.6	2.27 ± 0.06	2.01 ± 0.12
3	R	Pars triangularis	5.19	888	54.3	26.1	9.1	2.63 ± 0.09	2.32 ± 0.14
4	R	Lateral orbitofrontal	4.96	402	15.5	35.5	−24	2.24 ± 0.17	1.94 ± 0.11
5	R	Superior frontal	4.45	270	17.7	38.7	42.2	2.54 ± 0.08	2.27 ± 0.14
6	R	Pars orbitalis	3.92	728	40.2	51	−7.2	2.53 ± 0.16	2.13 ± 0.22
7	R	Posterior cingulate	3.84	94	9.7	−1.6	41.2	2.65 ± 0.18	2.36 ± 0.12
8	R	Postcentral	3.44	121	61.5	−9.7	30.5	2.46 ± 0.14	2.17 ± 0.16
9	R	Precuneus	3.44	88	7.4	−60.7	57.3	2.37 ± 0.16	2.11 ± 0.17
10	R	Rostral middle frontal	3.45	725	23.1	54.6	21.1	2.30 ± 0.16	2.05 ± 0.15
11	R	Superior temporal	3.41	263	51.6	−18.1	−5.4	2.77 ± 0.21	2.47 ± 0.15
12	R	Precentral	2.91	60	50.4	−0.1	41.4	2.72 ± 0.22	2.41 ± 0.17
13	R	Superior frontal	2.90	65	7.9	37.4	42.5	2.81 ± 0.19	2.51 ± 0.21
14	R	Caudal middle frontal	2.83	21	31	13.9	47.8	2.46 ± 0.12	2.25 ± 0.16
15	R	Superior temporal	2.73	29	49.8	6.3	−16.9	3.22 ± 0.33	2.74 ± 0.32

Each area consists of adjacent voxels showing a significant group difference; some brain structures have more than one area of change. The magnitude of the peak (t statistic) in each area and its Tailarach coordinates (a standardized common brain space) are listed, together with the size (in normalized space) and the mean and SD of the cortical thickness for the control and baseline ACLF groups

*L* left, *R* right

**Fig. 2:**
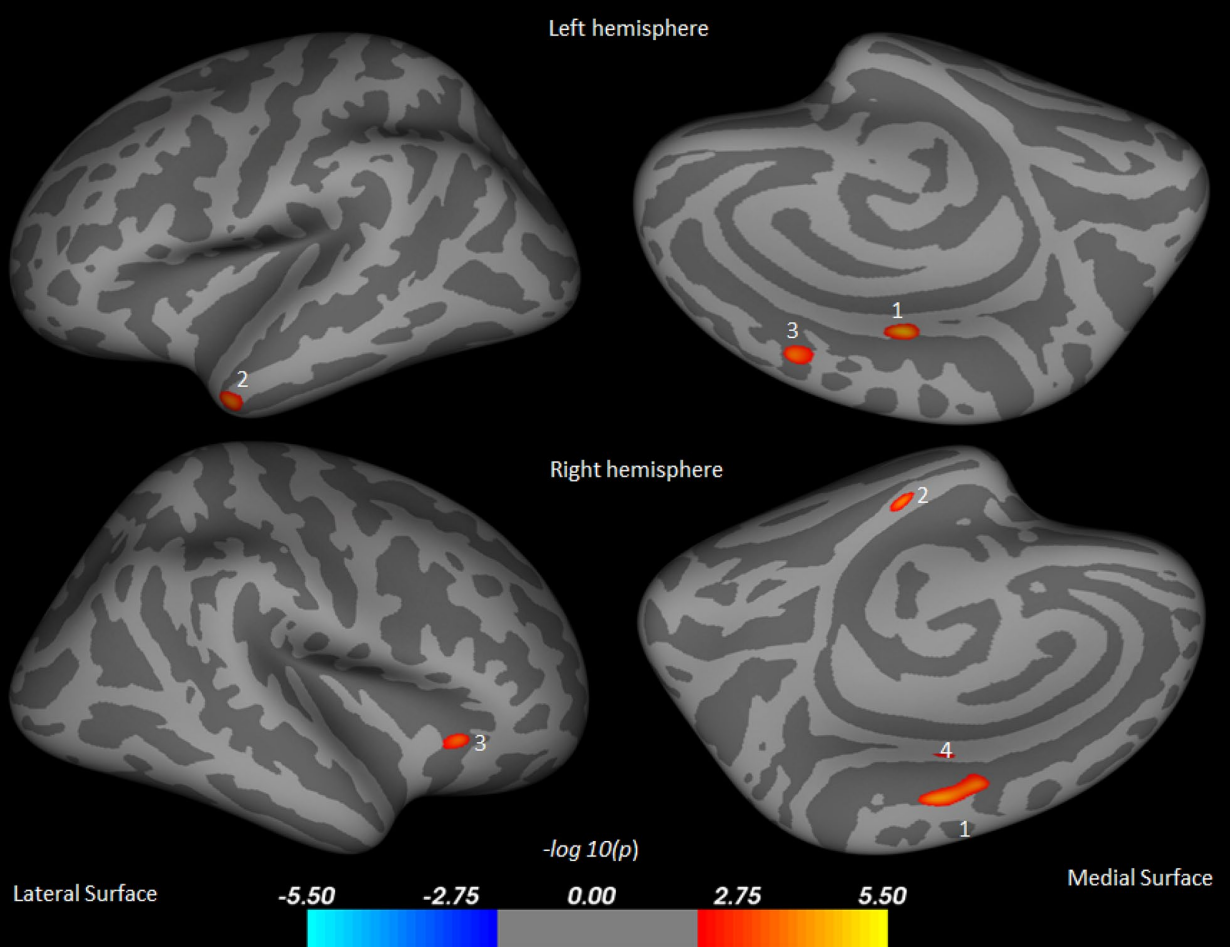
Brain regions showing significantly reduced cortical thickness in ACLF post recovery over control subjects in the left hemispheres overlaid on inflated pial surface, include posterior cingulate (*1*), middle temporal (*2*), and superior frontal (*3*) and right hemispheres include paracentral (*1*), parahippocampal (*2*), lateral orbitofrontal (*3*), and posterior cingulate (*4*)

At 3 weeks of clinical recovery, these patients still showed decreased cortical thickness in few brain sites as compared to those of controls (Fig. 2). These areas include posterior cingulate, middle temporal, superior frontal regions in the left hemisphere (Table 3; Fig. 2) and paracentral, parahippocampal, lateral orbitofrontal, posterior cingulate regions in the right hemispheres (Table 3; Fig. 2).

**Table 3:** Surface areas of significantly reduced CT in ACLF post-recovery over control and increased CT in post-recovery over baseline from both hemispheres

Cluster No.		Structures in area	Max t statistic	Size (mm^2^)	TalX	TalY	TalZ	Thickness (mm), mean ± SD	
								Control	Post ACLF
1	L	Posterior cingulate	5.392	79	−5.8	−13.9	39.2	2.77 ± 0.19	2.63 ± 0.16
2	L	Middle temporal	4.887	196	−45.6	3.8	−34.3	3.27 ± 0.23	2.99 ± 0.21
3	L	Superior frontal	4.768	62	−11	14	45.6	2.59 ± 0.16	2.54 ± 0.23
1	R	Paracentral	5.153	166	10.1	−15.5	48.9	2.44 ± 0.12	2.42 ± 0.21
2	R	Parahippocampal	4.928	59	25.2	−20.9	−27.3	3.20 ± 0.31	3.03 ± 0.22
3	R	Lateral orbitofrontal	4.671	50	30.4	28.9	−2.4	2.72 ± 0.12	2.62 ± 0.23
4	R	Posterior cingulate	3.926	17	5.1	−12.5	39.4	2.65 ± 0.13	2.64 ± 0.24

Cluster no.		Structures in area	Max t statistic	Size (mm^2^)	TalX	TalY	TalZ	Thickness (mm), mean ± SD	
								Baseline ACLF	Post ACLF
1	L	Lateral orbitofrontal	−2.745	577	−29	50.8	−10.9	2.08 ± 0.15	2.33 ± 0.15
2	L	Rostral middle frontal	−2.632	93	−37.1	43.4	0.3	1.95 ± 0.12	2.12 ± 0.11
3	L	Rostral middle frontal	−2.244	25	−38.6	26.5	35.6	2.25 ± 0.10	2.35 ± 0.09
4	L	Rostral middle frontal	−2.210	43	−33.9	32.4	29.5	2.06 ± 0.12	2.24 ± 0.14
5	L	Superior frontal	−2.204	59	−8.1	62.2	16.9	2.25 ± 0.16	2.50 ± 0.17
6	L	Lateral orbitofrontal	−2.013	4	−35.9	25.6	−17.7	2.31 ± 0.18	2.51 ± 0.12
1	R	Entorhinal	−3.97	185	21	−11.2	−29.2	2.89 ± 0.22	3.37 ± 0.22
2	R	Superior frontal	−2.313	71	8.3	58.1	26.5	2.29 ± 0.18	2.53 ± 0.15
3	R	Rostral middle frontal	−2.232	74	19.7	57	17.6	2.06 ± 0.21	2.30 ± 0.12
4	R	Lateral orbitofrontal	−2.086	36	17.1	39.4	−18.9	2.04 ± 0.14	2.26 ± 0.20
5	R	Pars triangularis	−2.0108	2	54.3	25.6	10.1	2.30 ± 0.14	2.50 ± 0.17

Each area consists of adjacent voxels showing a significant group difference; some brain structures have more than one area of change. The magnitude of the peak (t statistic) in each area and its Tailarach coordinates (a standardized common brain space) are listed, together with the size (in normalized space) and the mean and SD of the cortical thickness for control and post recovery ACLF group. And baseline and post recovery ACLF group

*CT* cortical thickness, *L* left, *R* right

**Fig. 3:**
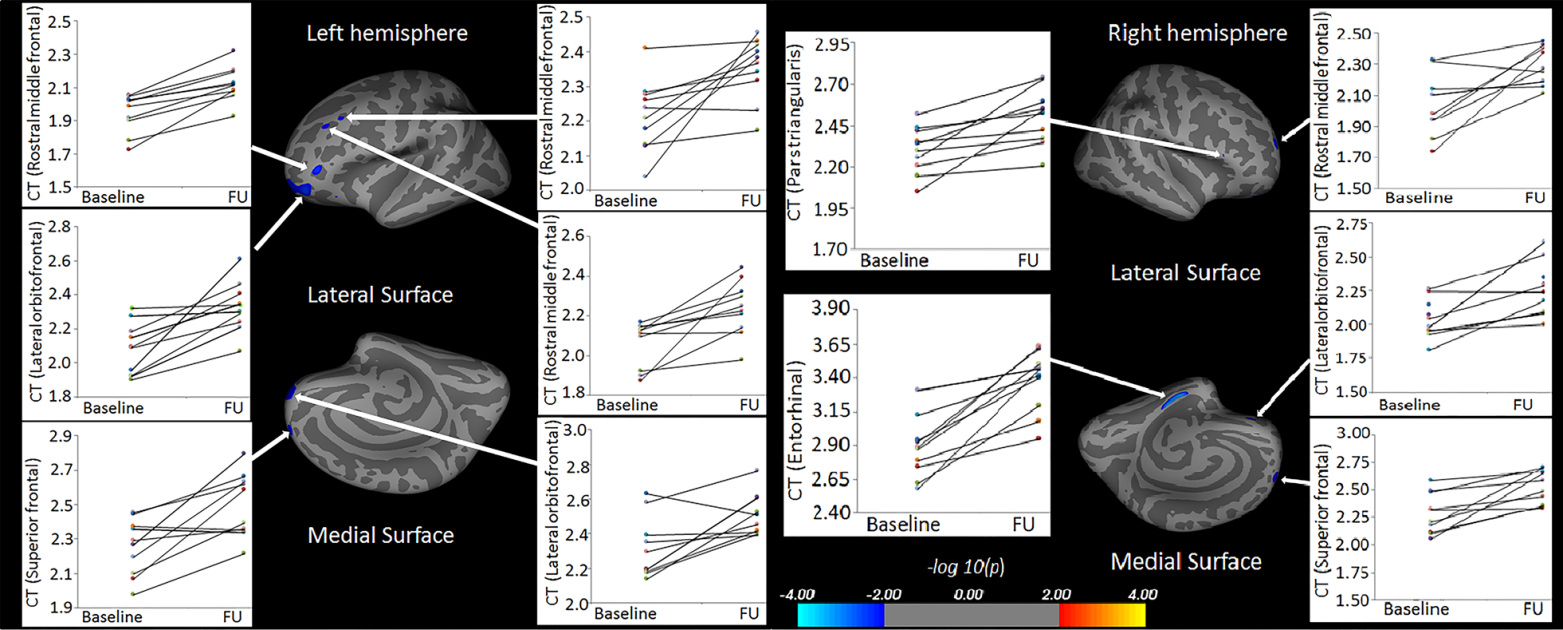
Brain regions with scatter plots showing significantly increased cortical thickness in post recovery compare to baseline in the left hemispheres overlaid on inflated pial surface, include lateral orbitofrontal, rostral middle frontal, rostral middle frontal, rostral middle frontal, superior frontal, and Lateral orbitofrontal and right hemispheres, include entorhinal, superior frontal, rostral middle frontal, lateral orbitofrontal, and pars triangularis

In follow-up study, significantly increased cortical thicknesses in lateral orbitofrontal, rostral middle frontal, rostral middle frontal, rostral middle frontal, superior frontal, lateral orbitofrontal regions in the left hemisphere (Table 3; Fig. 3) and in entorhinal, superior frontal, rostral middle frontal, lateral orbitofrontal, pars triangularis regions in the right hemisphere (Table 3; Fig. 3) were observed compared to those of baseline study. In follow-up study, our longitudinal FreeSurfer processing pipeline reduced the chances of positioning variation during data processing.

### Metabolites levels in controls, baseline and post recovery ACLF patients

At baseline, ACLF patients showed significantly decreased NAA (p = 0.001) and mI (p = 0.001), and increased Glx (p = 0.001) as compared to those of controls (Table 4). At 3 weeks clinical recovery, these patients still showed significantly decreased NAA (p = 0.004), and mI (p = 0.001), and significantly increased Glx (p = 0.030) levels than those of controls (Table 4). When compared to baseline metabolites level, only Glx level was significantly decreased (p = 0.024) in follow-up patients (Table 4).

**Table 4:** Mean and standard deviation of metabolites level in ACLF (baseline) compared to controls and follow−up

Groups	Metabolites (mmol/kg)		
	NAA	MI	Glx
Control (a)	12.9 ± 1.30	2.60 ± 0.50	15.0 ± 2.50
Baseline (b)	10.9 ± 1.15	1.40 ± 0.69	22.5 ± 5.41
Follow−up (c)	11.2 ± 1.00	1.72 ± 0.41	18.1 ± 3.62
p-values			
a vs b	a − b = 0.001	a −b = 0.001	a − b = 0.001
a vs c	a − c = 0.004	a − c = 0.001	a − c = 0.030
b vs c	b − c = 0.424	b − c = 0.184	b − c = 0.024

*NAA* *N*-acetyl aspartate, *mI* myo inositol, *Glx* glutamine/glutamate

### Neuropsychological findings in controls and post recovery ACLF patients

**Fig. 4:**
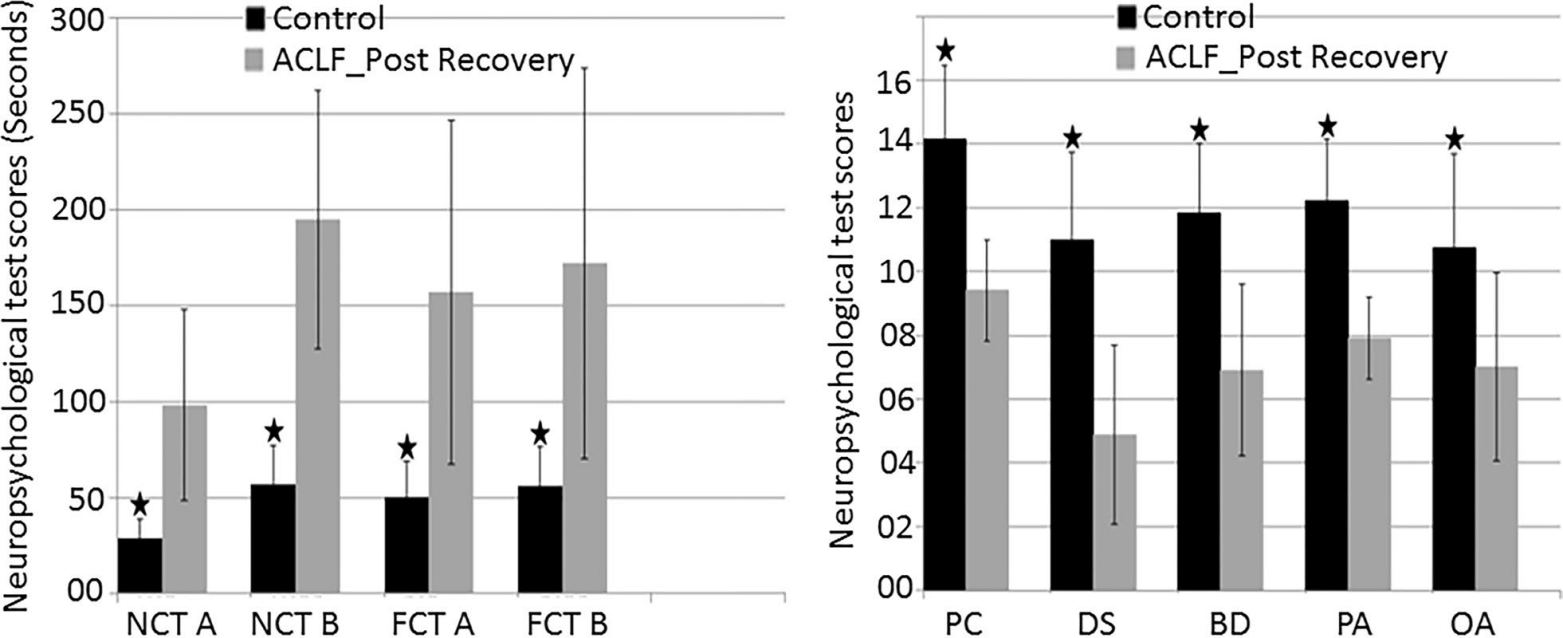
On *bar graphs* control subjects are showing significantly lower trail making test scores (NCT-A,B and FCT-A,B) and higher WAIS-P test scores (PC, DS, BD, PA and OA) as compared to post recovery ACLF after 3 weeks of conservative therapy

Neuropsychological tests were performed in controls and in follow-up ACLF patients after clinical recovery. Follow-up ACLF patients showed significantly higher NP tests score for trial making test (number connection test (NCT)-A, p = 0.002; NCT-B, p = 0.001; figure connection test (FCT)-A, p = 0.004 and p = 0.006) and lower NP tests score for Wechsler Adult Intelligence Scale (WAIS)-P (picture completion (PC), p = 0.001; digit symbol (DS), p = 0.001; block design (BD), p = 0.001; picture assembly (PA = , p = 0.001 and object assembly (OA), p = 0.003) compared to those of healthy controls (Fig. 4).

## Discussion

In the current study, decreased cortical thickness was detected in multiple brain areas in ACLF patients compared to those of controls, suggesting loss of gray matter tissues. These cortical areas regulate various cognitive, autonomic, language, visual sensory and motor functions that are deficient in ACLF patients. The pathological processes contributing to the altered regional cortical thickness may be due to increased cerebral hyperammonemia, proinflammatory cytokines and free radicals resulting from diminished liver function and/or secondary to infection. Reduced cortical thickness in patients with ACLF can be better explained by the ex vivo histopathological analysis. Though, no brain histopathological study is available on ACLF patients, while one brain histopathological study in cirrhotic patients with end-stage-liver disease with and without alcoholic etiology showed brain atrophy and Alzheimer’s type II astrocytosis [33]. We suggest that decreased cortical thickness in ACLF patients may have similar brain changes, a contention that needs to be validated on animal models of ACLF and that opens an inquisitive area of research.

The current findings in ACLF patients are consistent with the previous MRI finding in patients with liver disease associated with minimal HE and history of overt HE, which showed decreased gray matter density and cortical thickness in various brain sites [10, 13–15]. Chen et al., have showed decreased grey matter volume in many cortical areas in cirrhotic patients with a history of overt HE [10] and suggested that the presence of Alzheimer type II astrocytosis in gray matter and a diffuse spongy degeneration of cortex might be responsible for neuronal damage and lead to gray matter atrophy. Another study performed by Guevara et al., using voxel based morphometric analysis observed reduced regional gray and white matter areas in multiple brain sites including cingulate, precuneus, temporal, occipital lobes and precentral in cirrhotic patients [34]. Earlier, studies based on DTI and ^1^H MRS have showed significant abnormality in DTI metrics as well as metabolites level in patients with ACLF [18–21]. It has been suggested that the loss of neuronal and glial cells are responsible for these changes.

HE is a diffuse brain disease and influences all brain structures including both gray and white matter as well as deep brain structures such as basal ganglia [11, 13, 14, 16–19, 35]. Cortical structures and the limbic system structures (i.e. basal ganglia) interact via a relatively well understood and elaborate system of interconnections and damage to the basal ganglia can produce many of the same cognitive impairments as damage to the other cortical structures [36]. We suggest that any changes in metabolites levels in basal gangalia would also reflect the metabolites changes in other cortical areas. In the current study, spectroscopic information from basal ganglia is used as a proxy to explain the plausible mechanistic pathway for cortical thickness changes in ACLF patients. On ^1^H-MR spectroscopy ACLF patients showed significantly decreased NAA, and mI while increased Glx, as compared to those of controls [19]. Since NAA and mI are markers of neuronal and glial cells, respectively, the decreased concentration of these metabolites in ACLF suggests the contribution of decreased neuronal and glial cells density in cortical thickness loss. It has been reported that ammonia plays a key role in the pathogenesis of HE [10, 34]. Previous studies have demonstrated a correlation between altered brain metabolism and blood ammonia level in cirrhotic patients as well as in animal model [37–39]. Significantly increased brain Glx in ACLF patients, as observed in the current study as well as previous studies, reflects the increased ammonia level due to liver failure and this may initiate a cascade for the reduced cortical thickness in these patients. A study performed by Odeh et al., observed that TNF-α positively correlates with ammonia in patients with chronic liver disease [40] and supports the hypothesis of ammonia being a factor for the pathogenesis of HE [41, 42]. Cauli et al., have showed that proinflammatory cytokines may alter the blood brain barriers (BBBs) and influence the synthesis of nitric oxides in astrocytic cells, resulting in increased diffusion of ammonia into the brain and subsequent development of HE and brain tissue loss [43]. Recently, a number of studies have demonstrated that during HE, ammonia impairs BBBs permeability by generating excessive oxidative/nitrosative stress directly within the endothelial cells leading to activation of the matrix metalloproteinases and to increased paracellular passage of proteins [44, 45]. In ACLF patients significantly increased blood ammonia and inflammatory cytokines may synergistically responsible for the reduced cortical thickness in multiple brain sites. Further studies are required to explore the relationship between the regional reduction in cortical thickness, blood ammonia level and proinflammatory cytokines in these patients.

### Follow-up study

All the brain regions, which showed significantly decreased cortical thickness at baseline were tend to reversible in follow-up study. Only few brain regions showed significantly lower cortical thickness compared to those of controls. The other brain regions, which appear to be recovered, still showed non-significantly decreased cortical thickness in follow-up patients when compared to those of controls (data not shown). In post recovery group, partial recovery in NAA, and mI and significant recovery in Glx were observed compared to baseline, indicating a positive metabolic outcome in these patients in response to conservative treatment. However, compared to controls, they still showed significant differences in these metabolites levels. Incomplete normalization of ^1^H-MR spectroscopy profile in follow up study suggests that metabolic recovery lags behind the clinical recovery, and results also suggest that cortical thickness is a much more sensitive marker of clinical recovery.

Post recovery patients showed abnormal NP tests score as compared to healthy controls, suggesting persistence of abnormal cognitive functions which is consistent with previous study in overt HE [46, 47]. The abnormal NP test score in the follow-up patients can be explained based on the reduced cortical thickness in various brain regions, as well incomplete recovery of metabolites level. It has been proposed that systemic inflammatory response exacerbates the neuropsychological alterations induced by hyperammonemia and that the change in neuropsychological functions following induced hyperammonemia is greater in those with more severe inflammation [48]. Presence of reduced cortical thickness and impaired cognitive functions in post recovery as compared to control, demonstrates that brain dysfunction induced by HE persisted after clinical resolution and provided a basis for further evolution of the disease.

Thiamine deficiency has been shown to occur in patients with liver diseases due to impaired metabolism and storage, decreased liver cell mass, along with decreased intake and poor absorption that results from chronic congestion in the mesenteric venous system. It has been demonstrated that reduced thiamine level influence the brain microstructures in ACLF patients and recovered after oral supplementation of thiamine [20]. A study on rat animal model of thiamine deficiency showed recovery in brain neuronal damage following intraperitoneal supplementation of thiamine [49]. Since all the ACLF patients enrolled in the current study received oral supplementation of thiamine as a part of their conservative treatment we suggest that improved liver functions secondary to thiamine may be responsible for the recovery of cortical thickness and metabolietes levels in ACLF patients.

It is important to mention some of the limitations of the current study, which include limited number of subjects, short duration of follow-up period, and lack of quantification of thiamine and ammonia levels in the blood.

## Conclusions

Significantly reduced cortical thicknesses appeared in ACLF over controls in both brain hemispheres including frontal, parietal, temporal and occipital lobes. These multiple brain sites are involved in regulating various functions including cognitive, autonomic, language, visual sensory and motor functions. We suggest that neuronal loss and decreased cellular density due to increased cerebral hyperammonemia, proinflammatory cytokines and free radicals might be possible reasons for the reduced regional cortical thickness in these patients. Presence of reduced regional cortical thickness, altered metabolites and NP test scores in post recovery subjects as compared to controls suggest incomplete recovery over clinical characteristics. Further study in the animal model may be useful to assess this correlation, and may provide more accurate explanation for the reduced cortical thickness. Nevertheless, the current method can be easily implemented on clinical scanner to evaluate and monitor the brain tissue changes in patients with liver failure during the course of treatment.

## Abbreviations

ACLF: acute-on-chronic liver failure
MRI: magnetic resonance imaging
1H-MRS: Proton MR Spectroscopy
NP: Neuropsychological
NAA: *N*-acetyl aspartate
mI: myo-inositol
Glx: glutamate/glutamine
HE: hepatic encephalopathy
ICP: intracranial pressure
DTI: diffusion tensor imaging
TR: repetition time
TE: echo time
NEX: number of excitations
BBB: blood brain barrier
